# Transcranial Alternating Current and Random Noise Stimulation: Possible Mechanisms

**DOI:** 10.1155/2016/3616807

**Published:** 2016-05-03

**Authors:** Andrea Antal, Christoph S. Herrmann

**Affiliations:** ^1^Department of Clinical Neurophysiology, University Medical Center, 37073 Göttingen, Germany; ^2^Experimental Psychology Lab, Department of Psychology, Center for Excellence “Hearing4all”, European Medical School, Carl von Ossietzky University, 26111 Oldenburg, Germany; ^3^Research Center Neurosensory Science, University of Oldenburg, Oldenburg, Germany

## Abstract

*Background*. Transcranial alternating current stimulation (tACS) is a relatively recent method suited to noninvasively modulate brain oscillations. Technically the method is similar but not identical to transcranial direct current stimulation (tDCS). While decades of research in animals and humans has revealed the main physiological mechanisms of tDCS, less is known about the physiological mechanisms of tACS.* Method*. Here, we review recent interdisciplinary research that has furthered our understanding of how tACS affects brain oscillations and by what means transcranial random noise stimulation (tRNS) that is a special form of tACS can modulate cortical functions.* Results*. Animal experiments have demonstrated in what way neurons react to invasively and transcranially applied alternating currents. Such findings are further supported by neural network simulations and knowledge from physics on entraining physical oscillators in the human brain. As a result, fine-grained models of the human skull and brain allow the prediction of the exact pattern of current flow during tDCS and tACS. Finally, recent studies on human physiology and behavior complete the picture of noninvasive modulation of brain oscillations.* Conclusion*. In future, the methods may be applicable in therapy of neurological and psychiatric disorders that are due to malfunctioning brain oscillations.

## 1. Introduction

Brain oscillations have proven to play important roles in multiple perceptual, motor, and cognitive functions [1, 2]. In case of disturbed brain oscillations, neurological and psychiatric diseases can be the consequences [3, 4]. In order to establish a causal relationship between oscillations and cognitive functions as well as try to restore disturbed oscillatory activity, it is desirable to develop new methods that can externally modulate and control them. Noninvasive brain stimulation (NiBS) methods such a repetitive transcranial magnetic stimulation (rTMS) have been reported to modify brain oscillations (e.g., [5]). Indeed, it is well documented that the periodic electromagnetic force generated during rTMS can result in local entrainment of biologically relevant rhythms, mimicking frequency specific oscillatory activities triggered, for example, by cognitive tasks. More recently, two types of transcranial electric stimulation (tES) methods have been introduced that also could serve this purpose: transcranial alternating current stimulation (tACS) and transcranial random noise stimulation (tRNS) [6]. The aim of our paper is to review some of the relevant results that shed light on the physiological mechanisms behind these two methods.

When considering the physiological mechanism underlying tES, we have to introduce some basic terms at first. If the effect of tES is measured during the interval of electrical stimulation, it is considered an* online effect *and is most probably due to alterations of membrane voltage that facilitate or inhibit neural firing. If, on the other hand, an effect is measured after the end of the stimulation, it is considered an* offline effect *or* after-effect*. These are most probably induced by changes in synaptic activity/plasticity [7–9]. With regard to the measurements, in animal studies the outcomes are usually either neural spike rates or some form of coherence between the tES signal (e.g., a sine wave in case of tACS) and the neural signal. Typically, the stimulation intensity required to modulate a spike rate is higher than that required to modulate coherence [10]. In human studies, the measures are commonly either behavioral (reaction times, performance, etc.) or physiological, that is, obtained from the electroencephalogram (EEG), magnetoencephalogram (MEG), or electromyogram (EMG). A standard procedure for quantifying the effects of a tES technique is to measure the magnitude and time course of its effects on primary motor cortex (M1) excitability, by quantifying single pulse TMS-elicited motor evoked potentials (MEPs) in EMG recordings [11]. Since this is a very straightforward procedure relying on an objective outcome parameter, a high percentage of the tES studies, including tACS and tRNS applications in healthy subjects, were carried out using M1 as a model.

## 2. Transcranial Alternating Current Stimulation

### 2.1. Shifting the Resting Potential of the Membrane Voltage: Comparison to Direct Current (DC) Stimulation

**Figure 1:**
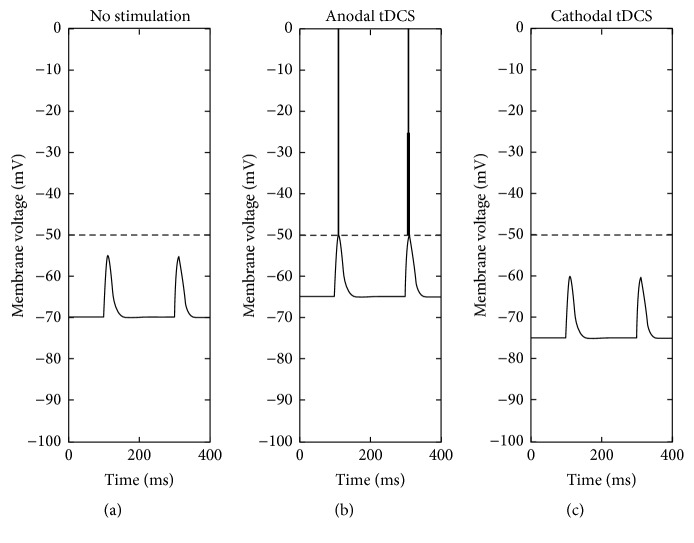
Assumed neural mechanism of tDCS. (a) Without tDCS, the resting potential of the cell is at −70 mV and an incoming excitatory postsynaptic potential (EPSP) arriving 100 ms after onset of the experiment does not reach the threshold for firing at −50 mV (dashed line). (b) If the neuron is close to an anode, the positive voltage from the anode will raise the resting potential towards a more positive voltage and the same EPSP will exceed the threshold and result in a neural spike. (c) If the neuron is close to a cathode, the negative voltage from the cathode will lower the resting potential towards a more negative voltage and the same EPSP will not exceed the threshold.

With regard to DC stimulation one of the questions that was first addressed in early animal studies was whether externally (transcranially or subdurally) applied electric fields are able to modulate neuronal activity. It was shown that anodal stimulation could enhance neural firing rates while cathodal stimulation resulted in a reduction of the firing rate [12, 13]. The mechanism behind this differential modulation of cortical excitability is believed to rely on up- or downregulation of the resting potential of the neurons' membrane voltage in response to anodal or cathodal stimulation, respectively (Figure 1) [14–16]. The effects of transcranial direct current stimulation (tDCS) are always subthreshold, meaning that the polarization induced when a neuron is silent will not be able to drive the generation of action potentials. Evidently, weak polarizations can modulate the probability of action potentials when a neuron is activated by other neuronal input.

Neuronal compartments are polarized in different ways during stimulation, with the soma typically polarized in opposite way than dendrites [16, 17]. Thus, a stimulation considered anodal (depolarizing) for the neuronal soma (which can control the generation of action potentials very directly) is cathodal (hyperpolarizing) for the dendrites and vice versa. In the case of DC stimulation the somatic polarization probably plays the dominant role in the development of the online effects. During AC stimulation, the dendritic polarization could also be relevant, since brain oscillations can determine how neurons process the sometimes opposing synaptic inputs.

In human studies, a bipolar stimulation of neuronal networks can be induced by placing both anodal and cathodal electrodes on the scalp. However, the current flow is not always maximal directly beneath the stimulation electrodes but rather at their rims under the connectors and between electrodes [18]. In case only one of the two mechanisms (excitation* or *inhibition) is required, one of the electrodes can be placed extracranially [19]. Alternatively, a small stimulation electrode can be combined with a larger “passive” return electrode. This procedure leads to strong current densities under the small stimulation electrode and weaker current densities under the large return electrode [20].

One of the main technical differences between the more established tDCS and the more recent tACS is the functional interpretation of the two or more electrodes that have to be mounted on the scalp. In case of tDCS, as it was mentioned above, these two electrodes are referred to as anode and cathode and result in excitation or inhibition of the targeted underlying cortex, respectively. For tACS the concept of how to modulate brain oscillations is quite different due to the applied alternating current. During one half cycle of a tACS oscillation, one electrode will serve as anode and the other one as cathode and current strength will increase and decrease following a half sine wave. During the other half cycle, the pattern will reverse and the former anode must now be considered the cathode and vice versa. Thus, on average, the membrane potential is not affected and therefore tACS is probably not so well suited to enhance or decrease excitability of cortex over sustained intervals of time as is tDCS. However, the effects during the brief depolarizing or hyperpolarizing phase of each half cycle could already be strong enough to induce online effects via entrainment, an effect discussed below.

## 3. How Can tACS Influence Cortical Activity?

### 3.1. Computer Models Can Predict Intracranial Current Densities

A common question about tES is whether a weak extracranially applied current is able to influence the activity of cortical neurons in the human brain, which in turn alters cognitive functions. We will review a series of studies that have addressed this issue in modelling tDCS current flow. In principle, many of the basic tDCS findings should also hold for tACS [21]. The maximum current flow is, of course, only reached during the peak of the alternating current and changes polarity during the two half-waves, that is, the direction of current flow changes by 180°. Computer simulations of the current flow during tDCS using human head models have revealed that a significant amount of the current is shunted by the well-conducting skin (~90%), while much less of the current actually reaches the brain [22]. In the case of tACS, the different frequency response of each type of conducting element between the electrodes and the brain should also be taken into account [23].

Miranda et al. [24] demonstrated in an isotropic spherical head model that 2 mA of tDCS results in an intracranial current density of 0.1 A/m^2^ (Ampere per square meter). Using a value of 0.450 S/m (Siemens per meter) for the conductivity of the brain, the authors argued that this results in an electric field gradient of 0.22 V/m (Volt per meter). In a more recent article these authors used a more realistic head model derived from magnetic resonance imaging (MRI) measurements resulting in a maximum electric field of 0.38 V/m using a gray matter conductivity of 0.33 S/m [24].

**Figure 2:**
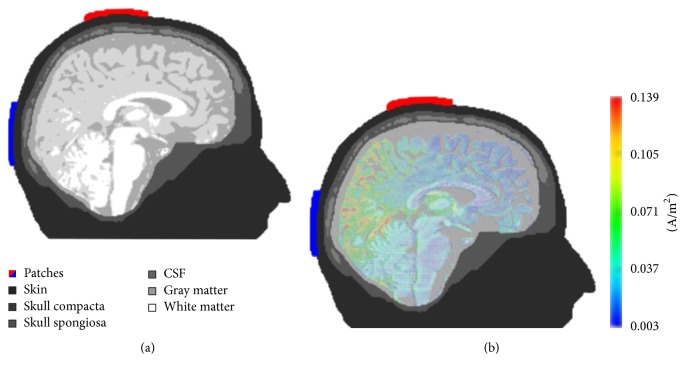
(a) Anisotropic finite element model for simulations of intracranial current flow. Stimulation electrodes are at the EEG electrode positions Cz and Oz of the international 10–20 system for electrode placement (red and blue, resp.). (b) Current density simulations reveal strongest current flow is in the posterior part of the brain underneath and between the stimulation electrodes. Reprinted with permission of the authors from [25].

Neuling et al. [25] used a very fine-grained anisotropic finite element model of the human head to show that 1 mA of tDCS/tACS applied to human visual cortex results in intracranial current densities of 0.05 to 0.15 A/m^2^. This amounts to a cortical electric field of 0.417 V/m when using the mean current density of 0.1 A/m^2^ if a gray matter conductivity of 0.24 S/m is assumed (Figure 2). Note that in this paper we assume gray matter conductivities in the range from 0.333 to 0.352 S/m, while the above-mentioned articles used values in a wider range from 0.24 to 0.45 S/m.

**Figure 3:**
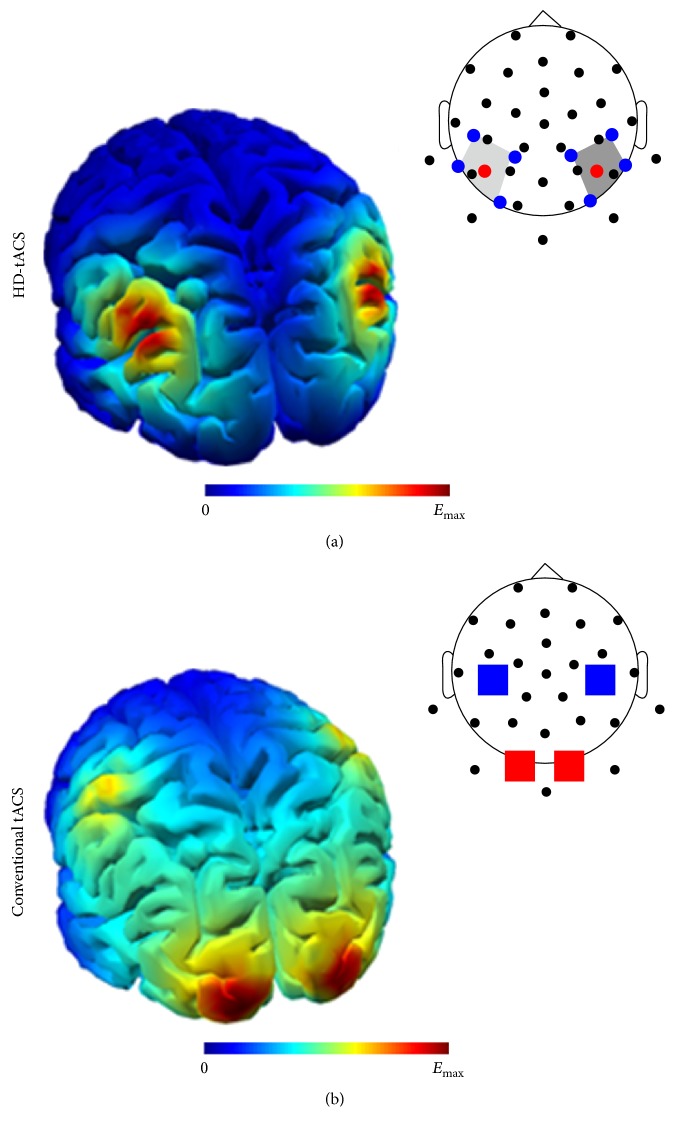
Different montages result in different patterns of current densities. (a) tACS with two 4 × 1 electrode montages (high density, HD) was used in order to achieve a more focal current density. (b) Trying to achieve a similar pattern of current flow with conventional large electrodes results in a more widespread distribution of currents. Reprinted with permission of the authors from [27].

The above-mentioned studies used two conventional large stimulation electrodes with an area of approx. 35 cm^2^ per electrode. Large area under the electrode is desirable to reduce the current density in the skin. Using multiple smaller electrodes yields stronger intracranial current densities (Figure 3). Together with an optimized placement of the electrodes derived from simulations this can significantly enhance intracranial current densities and result in a more focal stimulation [26].

The above-mentioned studies compute intracranial current densities that allow the prediction of the location and intensity of tES-effects. However, this is not sufficient to answer the question, whether or not the computed intensity is below or above the threshold to elicit neuronal spiking in neurons that would not spike without these extracranially applied currents. In order to come to a conclusion about this topic, we need to consider animal studies with intracranial recordings.

### 3.2. Animal Studies

As was mentioned above, the application of very weak electric fields applied to the human brain extracranially leads to the question, what intensity of stimulation is required to see an effect upon neural activity. In animal studies this issue is frequently addressed; however, it is still not clear how these data can be transferred to human studies. Indeed, the stimulation protocols applied in animal studies are often quite different from those used in humans. Therefore, these data should be handled with caution. Furthermore, knowing the magnitude of the electric field inside the brain is not enough to estimate the effects on neurons. The geometry and orientation of neurons relative to the electric field determine the amount of polarization [28]. Neurons oriented parallel to the electric field polarize maximally while neurons oriented perpendicularly are ideally not polarized at all. Currents in the dendrites-to-axon direction depolarize the soma, while axon-to-dendrites currents hyperpolarize it. Considering the convolution of the cortex, currents flowing into a gyrus are expected to polarize neurons on one side with a given polarity but neurons of the other side of the gyrus might react in the opposite way. Therefore, at the single neuron level, the definition of “anodal” versus “cathodal” stimulation is less obvious if the orientation relative to the electric field and geometry of the neuron is not specified. The fact that anodal/cathodal stimulation usually increases/decreases excitability is probably due to a higher number of depolarized/hyperpolarized neurons. Furthermore, the dynamics of the stimulated populations of neurons can rectify the effects of electric fields as was shown using a computational model [29]. Nevertheless, a clear experimental validation of this issue is still lacking.

Francis et al. [30] were able to demonstrate that electric pulses of 140 *μ*V/mm root mean square or 295 *μ*V/mm peak amplitude (0.295 mV/mm) are sufficient to increase the firing rate of single neurons in hippocampal slice preparations from the rat. However, these pulses resemble neither tDCS (constant current typically for multiple minutes) nor tACS (sine wave for multiple minutes) closely. Reato et al. [31] performed electrical stimulation experiments in slices of rat hippocampus and network simulations. Both experiments revealed a threshold of 0.2 mV/mm before an electrical AC was able to modulate ongoing neural activity.

A more direct comparison of animal findings to tACS is possible for the application of electric fields of alternating currents (AC) to cortex. Fröhlich and McCormick [10] applied such AC fields to the cortical slices of ferrets. They were able to demonstrate that AC fields as low as 0.5 mV/mm were sufficient to modulate ongoing neural activity. In a simulation study with a spiking neuronal network, the authors showed that at low stimulation intensities only spike timing was modulated but spike rate was not altered [31]. In this case, spikes would align to certain phases of the externally applied sine wave. At higher intensities, also the spike rate was modulated.

A further question is how much of an extracranially applied electric current actually arrives at the cortex. Ozen et al. [32] attached stainless steel wires to the skull of anesthetized rats, stimulated them electrically with AC, and simultaneously recorded intracranial neural activity. They were able to demonstrate an entrainment of ongoing neuronal activity at frequencies mimicking the frequency of cortical slow oscillations (0.8–1.7 Hz) in multiple cortical areas. The authors reported that membrane potentials as well as unit activity were modulated by AC stimulation. The experiments revealed that voltage gradients of 1 mV/mm in the extracellular space were sufficient to affect discharge probability of neurons.

### 3.3. Tissue Resistivity/Conductivity

A last step that is required before tES intensity can be compared to neural firing or phase-locking thresholds is to determine the gray matter resistivity or conductivity, which is the inverse of resistivity. The intensity of tES is specified in current strength in the unit A (Ampere) or mA (milliAmpere, i.e., 1/1000 Ampere). As we have seen, modelling studies can predict intracranial current densities from these extracranial current strengths. Current density is given in A/m^2^ (Ampere per square meter). Animal studies apply a voltage across the cortex in order to determine the threshold for modulating neural activity. Two electrodes are required to do so; therefore the resulting unit is a voltage gradient specified in V/m (Volt per meter); that is, 500 *μ*V could be applied at two electrodes spaced 1 mm apart resulting in 500 *μ*V/mm which converts into 0.5 mV/mm or 0.5 V/m. In order to convert current density into voltage gradients, the resistance of the brain is required according to Ohms law: *U* = *R∗I* where *U* is voltage, *R* is resistance, and *I* is current flow.  Table 1 gives an example of such a conversion for 1 mA of tES intensity.

The resistivity of tissue is typically measured in animals and it is assumed that it is of equal value in humans. Measurements in rabbits and cats resulted in values for tissue resistivity of 2.84 Ωm (Ohm meter, corresponding to a conductivity of 0.352 S/m) for gray matter at low frequency and body temperature [33]. However, many researchers use values that slightly differ, for example, 3.03 Ωm (conductivity 0.333 S/m), for inverse modelling [34].

**Figure 4:**
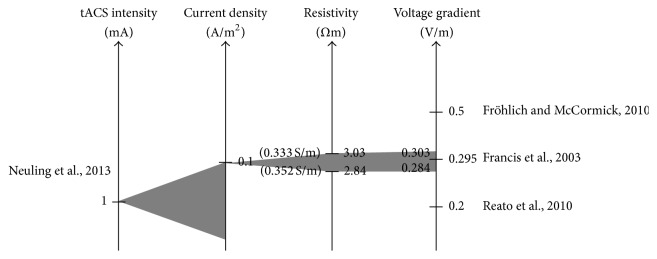
Translation of tACS intensity to intracranial voltage gradients allows a comparison to thresholds for eliciting spikes in animal research. The left axis represents tACS intensity. Neuling et al. [35] applied 1 mA intensity (peak-to-peak value: translates to a sine wave of 0.5 mA amplitude). In their FEM, they could show that this intensity results in a number of current densities in different parts of the brain with a maximum of 0.1 A/m^2^ (axis: current density). The third axis represents the tissue resistivity of gray matter (tissue conductivity given on the left of the axis in brackets). Values in the range from 2.84 to 3.03 Ωm result in voltage gradients from 0.284 to 0.303 V/m being in the range of thresholds for neural firing or phase-locking (axis: voltage gradient). Note that for voltage gradients 1 mV/mm is equal to 1 V/m.

With this value we finally have all the information needed to convert a tES intensity into an intracranial voltage gradient.  Figure 4 demonstrates this procedure; for example, Neuling et al. [25] applied tACS at an intensity of 1 mA. They were able to show that this stimulation intensity results in intracranial current densities up to 0.1 A/m^2^ [21]. Note, however, that a range of current densities was found in the brain as represented by the gray area in the figure. Multiplying this current density with the gray matter resistivity of 2.84 Ωm results in an intracranial voltage gradient of 0.284 V/m. Using 3.03 Ωm instead would result in a voltage gradient of 0.303 V/m.

### 3.4. Comparing tES to Neural Thresholds

Comparing the above-mentioned values for voltage gradients resulting from tES to the above-mentioned thresholds for neural firing or phase-locking demonstrates that 1 mA of tES would be above the lowest threshold determined by, for example, Reato et al. [31]. However, for the other thresholds that have been defined, tES seems to be near or below threshold (Figure 4). Due to the wide range of thresholds observed in animal experiments, it can be concluded that tES seems to result in voltage gradients that are roughly within the range of thresholds as obtained from animal experiments. However, despite the differences of the experimental preparations, some forms of entrainment using electric fields were reported in many AC studies, suggesting the entrainment may be a generic way electric fields can affect neuronal networks. The idea of entrainment of neuronal oscillations by weak electrical alternating current stimulation was first introduced by Deans et al. [36] for induced gamma oscillations. At the single neuron level [37] showed how weak AC polarization entrained neuronal firing and modulated spike timing. Modulation of spontaneously active Purkinje cells by AC fields was also already shown by Chan et al. [15]. In later studies Fröhlich, Ozen, and Reato extended these concepts for slow-waves and gamma oscillations. The values of the coupling constant (how many mV a neuron polarizes per V/m electric field) measured in many of the studies were highly comparable. A value of 0.1–0.2 mV/V/m for the somatic polarization has been measured in [10, 16, 28, 36, 37]. The existence of such a reliable value suggests that the sensitivity of single neurons is always more or less the same across experimental preparations and maybe species. The different thresholds found in animal preparations could be explained by the differences in the dynamics underling the neuronal networks of interests.

The fact that behavioral results are obtained in well-controlled human experiments suggests that probably the lower of the thresholds of Figure 3 is to be taken into consideration for tES. Future experiments in humans are required, for example in patients with intra-cranial recording electrodes.

### 3.5. The Most Relevant Factors for Applying tACS in Human Studies

As mentioned above, compared to tDCS a different mechanism is at work during tACS, requiring a different rationale for designing an experiment. At first, experimenters should identify a cognitive process that is characterized by a specific brain oscillation or combination of oscillations. Next, what parameter of this brain oscillation is responsible for which aspect of the cognitive process has to be defined. In principle, three key parameters as well as higher order interactions can be taken into consideration.

(1) The amplitude of brain oscillations has been shown to be correlated with cognitive functions. For example, EEG alpha oscillations have been closely linked to visual perception [38] and different amplitudes of this oscillation determine whether or not a near-threshold visual stimulus will be consciously perceived by human subjects [39].

(2) The frequency of brain oscillations is also crucial for cognitive functions. For example, the number of gamma cycles (30–80 Hz, 25 ms cycle length at 40 Hz) that fit into one theta cycle (4–8 Hz, 125 ms cycle length at 8 Hz) is believed to determine the number of items that human subjects can keep in short term memory, that is, 5 items (5*∗*25 ms) at a frequency relation of 40 Hz/8 Hz [40].

(3) The phase of brain oscillations affects cognitive functions, too. For example, the phase of delta oscillations is important for recognition of speech [41]. In addition, phase-locking or phase coherence between cortical sites is supposed to reflect neural teamwork. In line with this assumption, for example, phase-locking in the gamma frequency range between hemispheres is enhanced during perception of horizontal motion which requires interhemispheric integration [42].

In addition, higher order interactions between tACS and brain oscillations have to be taken into account. If we want to modulate brain oscillations by tACS, it will not be a linear process and the effect will not be limited to the frequency of stimulation. First, linear increases in stimulation intensity may have nonlinear effects on the affected neural tissue. Second, entraining oscillations does affect oscillations not only at the frequency of stimulation but also at harmonic multiples as well as subharmonics. In addition, it is known that certain frequencies interact with each other during cognitive processes, a process referred to as cross-frequency coupling [43]. Therefore, it has to be assumed that entraining one frequency may have effects also at other frequencies.

In the following, we do not intend to give a complete review of all tACS studies that have been carried out until now. That has been done recently (e.g., [44]). Instead, our goal is to focus on experiments that exemplify the above-mentioned parameters of modulating brain oscillations.

### 3.6. Modulating the Amplitude of Brain Oscillations with tACS

The most prominent oscillation in the human EEG is the alpha rhythm with a frequency in the range from 8 to 12 Hz. If the posterior part of the brain is stimulated with 10 Hz tACS this results in an enhancement of the EEG alpha amplitude during stimulation [45]. This after-effect can be seen for at least 30 minutes after 10 minutes of stimulation [35]. Such elevations of amplitudes of EEG oscillations have also behavioral relevance. For example, elevating the naturally occurring EEG delta oscillations (0–4 Hz) during sleep with 0.75 Hz tACS resulted in enhanced retention of declarative memories from the previous day [46]. Along the same lines, amplifying gamma oscillations (30–80 Hz) with 40 Hz tACS during sleep leads to the induction of lucid dreaming [47].

### 3.7. Modulating the Frequency of Brain Oscillations with tACS

Animal experiments (see above) have shown that stimulating cortical tissue with AC currents at a stimulation frequency below the frequency of intrinsic oscillations can slow down these brain oscillations [10]. When the stimulation was applied at a frequency above that of the intrinsic oscillations, the frequency of the brain oscillations was speeded up. This resembles the so-called entrainment of an oscillator, known from physical systems [48–51]. A similar effect was observed recently by Helfrich and coworkers [45]. These authors found a sharpening of the EEG alpha peak in the frequency spectrum during 10 Hz tACS over the visual cortex in humans. Along similar lines, tACS was used to slow down the frequency of human theta oscillations in order to improve short term memory capacity, since according to the model of Lisman and Spruston [52] slower theta oscillations can host more cycles of faster gamma oscillations [53].

**Figure 5:**
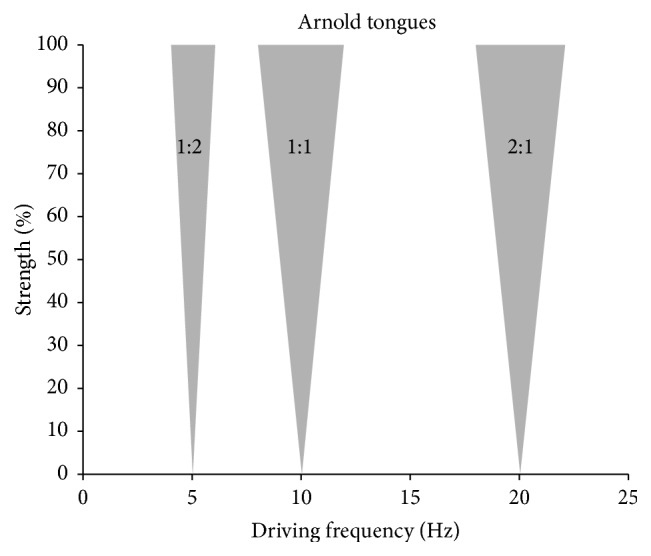
Theory of entrainment. If the brain is stimulated near its “Eigenfrequency,” that is, the individual alpha activity around 10 Hz, the EEG will synchronize to the frequency of the driving force (e.g., tACS). This is considered synchronization or entrainment of an oscillator by an external driving force and depicted in gray (1 : 1 region). If, however, the stimulation frequency is far from the “Eigenfrequency,” the EEG will be dominated by its “Eigenfrequency” (white regions of diagram). If the strength of the external driving force (tACS) increases, the synchronization regions will become wider in frequency. Due to this triangular shape the synchronization region is referred to as an Arnold tongue [51]. Synchronization can also happen at harmonics (*N* *∗* Eigenfrequency) and subharmonics (Eigenfrequency/*N*) where *N* is an integer (1 : 2 and 2 : 1 show here). They do not need to have the same shape and width.

Behaviorally, such frequency changes can modulate the temporal window for integrating auditory and visual input into one coherent percept [54]. Whether tACS can modulate the frequency of an ongoing brain oscillation, that is, entraining an oscillation, depends upon both the frequency and the amplitude of the stimulation. This can be visualized by the so-called Arnold tongue (Figure 5; see, e.g., [51]). If the frequency of tACS is very close to the frequency of intrinsic brain oscillations, even very low currents can influence the oscillations amplitude, phase, and frequency.

### 3.8. Modulating the Phase/Phase Coherence of Brain Oscillations with tACS

Modulating the alpha rhythm in the auditory cortex at 10 Hz revealed that the phase of the alpha activity determines whether or not a near-threshold auditory stimulus will be perceived [55]. Modulations of the interhemispheric phase coherence in the gamma band via 40 Hz tACS have led to altered perceptions of ambiguous motion stimuli [27, 56].

### 3.9. Modulating Cross-Frequency Coupling by tACS

Animal studies have demonstrated that entraining brain oscillations does affect oscillations not only at the frequency of stimulation (e.g., 10 Hz) but also at harmonic multiples (20 Hz, 30 Hz, 40 Hz, etc.) as well as subharmonics (e.g., 5 Hz) [57]. In humans, it has been shown that stimulating the brain with 40 Hz tACS reduces oscillatory power at 10 Hz representing cross-frequency coupling and supporting the known antagonism between gamma and alpha [27].

Due to the above-mentioned mechanism of tACS, we recommend a list of questions and suggestions for good practice in designing tACS experiments as follows.

*Good Scientific Practice for Planning a tACS Experiment. *Consider the following

 Which cognitive or motor process shall be modulated?
 Which brain oscillation is associated with this cognitive or motor process?
 Which parameter of the brain oscillation (amplitude, frequency, phase angle, coherence between two regions, etc.) is to be modulated to achieve the desired change in cognitive or motor processing?
 Ideally, the observed or desired effect should be modelled in a neural network. While it is relatively straightforward to model whether an incoming sensory stimulus will exceed the firing threshold of a neuron with a sinusoidally modulated resting potential, it is harder to find good models that can explain human reaction times or hit rates in models composed of multiple firing neurons.
 Which brain region should be targeted and which electrode montage is suited to achieve it?
 Model the intracranial current densities that result from tACS or refer to existing modelling results.
 Demonstrate both behavioral and physiological changes to ascertain the correlation of the two.
 Choose a plausible control condition to demonstrate that the observed effects are due to stimulation of a specific brain region at a specific frequency and so forth.
 Good care has to be taken in order to rule out that subjects can differentiate between stimulation and control conditions.

## 4. Transcranial Random Noise Stimulation

tRNS is a special form of tACS. During tRNS a low intensity alternating current is applied where intensity and frequency of the current vary in a randomized manner. The stimulation is biphasic. Like with tACS, various forms of noise may be applied, depending on the frequency ranges. In most of the studies using tRNS, a frequency spectrum between 0.1 Hz and 640 Hz (full spectrum) or 101–640 Hz (high-frequency stimulation) were used [58, 59]. The probability function of the noisy current stimulation follows a Gaussian or bell-shaped curve with zero mean and a variance, for which 99% of all generated current levels are within ±1 mA. In the frequency domain all coefficients of the random sequence have a similar amplitude (“white noise”).

tRNS over M1 had an effect comparable to the previously determined effect of anodal tDCS on the development of MEPs over time, enhancing the cortical excitability of this cortical area [59–62]. For example, Terney and colleagues [59] have shown that 10 minutes of tRNS applied over M1 with 1 mA intensity can induce facilitatory after-effects lasting up to 1–1.5 hours and is capable of improving the performance in an implicit motor learning task. It was also reported that the high-frequency subdivision between 100 and 640 Hz of the whole spectrum is functionally responsible for alteration of excitability in M1, superiorly to low frequency (0.1–100 Hz) stimulation. In another study M1 stimulation using tRNS enhanced motor skill learning compared to sham stimulation [63]. Compared to the time course of anodal tDCS, tRNS exerted more gradual effects, while tDCS resulted in large skill gains immediately following the onset of stimulation. Interestingly, the after-effect of tRNS is intensity dependent. Lower intensity stimulation at 0.4 mA tRNS leads to inhibitory after-effects comparable to what has been observed with cathodal tDCS using 1 mA or 140 Hz tACS using 0.4 mA [61]. This suggests that inhibitory neurons in M1 might have lower thresholds, at least for this kind of stimulation. On the behavioral level, effects of high-frequency tRNS were also demonstrated, for example, by Fertonani et al. [58]. In this study the authors applied tRNS to the visual cortices of healthy subjects during the performance of an orientation-discrimination task. A significant enhancement in visual perceptual learning during the application of high-frequency tRNS was observed compared to anodal and cathodal tDCS as well as sham stimulation. Interestingly, anodal tDCS induced a larger facilitation if it was applied before task execution and tRNS if it was applied during the task [64], suggesting that the ideal timing of application of different electrical stimulation methods varies and depends on the stimulation type. tRNS over the lateral occipital cortex facilitated facial identity perception [65]. In contrast, tRNS to the right dorsolateral prefrontal cortex (DLPFC) impaired categorical learning in a prototype distortion task [66]. These results demonstrate that depending on the involved cortical area and the type of protocols, tRNS can induce long-term positive but also negative changes of cognitive and brain functions. However, a neutral effect was also reported. With regard to the effect of tRNS on working memory performance, a study showed no effect of stimulation over the DLPFC on performance [67].

The physiological mechanisms of tRNS are not clarified completely yet. Animal studies on tRNS that could elucidate the effects of this technique are completely missing. Although higher frequencies (e.g., 140 Hz) have been shown to modulate brain activity, the neuronal membrane acts as a low-pass filter; therefore, high frequencies that are applied by tRNS are supposed to polarize neurons by a very small amount. Deans et al. [36] measured the polarization of neurons during AC stimulation and estimated the coupling constant between electric field and induced polarization (mV per V/m applied). They found that 100 Hz AC stimulation gives a coupling constant of 0.050 mV/V/m. Therefore, 1 V/m (max) in the brain at 100 Hz can polarize a neuron by only 50 *μ*V. This value appears very small with respect to significantly modulated brain function. However, as suggested by other studies, the stimulation of many synaptically connected active neurons can provide an amplification mechanism [10, 31].

One potential online effect of tRNS might be associated with repetitive opening of Na^+^ channels, as was observed in a study investigating the application of AC stimulation to rat hippocampal slices [68]. Along this line, in a recent pilot study the Na^+^ channel blocker carbamazepine showed a tendency towards inhibiting MEPs 5–60 minutes after stimulation [69]. Interestingly, the partial NMDA receptor agonist D-cycloserine, the NMDA receptor antagonist dextromethorphan that could block the effect of tDCS, had no significant effect on the excitability increases seen with tRNS.

Besides this, the effects of tRNS might be based on other mechanisms, such as stochastic resonance [70]. Briefly, stochastic resonance refers to the phenomenon that a signal that is too weak to exceed a threshold is amplified by adding noise, for example, when a neural oscillation in the brain is subthreshold. These, probably synaptically operated subthreshold activities, driven by oscillatory inputs that neurons receive from other brain regions, are not strong enough to induce action potential generation. If random noise is added, the sum of the two signals exceeds the threshold at certain times. The frequency of the suprathreshold signal is determined by the existing subthreshold neural oscillation. It was suggested that tRNS may increase synchronization of neural firing through amplification of subthreshold oscillatory activity, which in turn reduces the amount of endogenous noise. The improvement of the signal-to-noise ratio in the central nervous system and the sensitization of sensory processing can lead to enhanced perception or cognitive performance [71–73]. However, it is not clear how this process can induce long-term changes in the human brain [74, 75]. A study reported that bifrontal application of tRNS for 5 days enhanced the speed of both calculation- and memory-recall-based arithmetic learning [74]. Six months later the behavioral and physiological modifications in the stimulated group relative to sham controls were still present. Similarly, in another study repeated bilateral parietal stimulation increased numerosity discrimination ability [75] with an after-effect for several weeks.

## 5. Summary

Both subtypes of tES, tACS, and tRNS are effective at modulating neural brain activity and result in behavioral effects in animals and human subjects. They are increasingly used in the research and also in phase II clinical studies; for example, for the reduction of the symptoms in tinnitus patients it has been shown that tRNS is more effective than either tDCS or tACS [76]. They have a better blinding potential with regard to the cutaneous sensations, such as itching, tingling, or burning, compared to tDCS applications [77]. Nevertheless, phosphene perception during tACS in a wide frequency range (6–70 Hz) might affect the interpretation of results (e.g., by inducing shifts in attentional state/arousal).

Unfortunately, it is not completely clarified how these stimulation methods act on the neuronal level. The regular sinusoidal ups and downs of tACS result in a weak modulation of the membrane voltage. If neuronal input from other cells is just below threshold, this regular sinusoidal modulation may be sufficient to drive the neuronal input to exceed the threshold at the frequency of tACS. Similarly, the random fluctuations of external voltage in case of tRNS may be sufficient to help an otherwise subthreshold neural oscillation to exceed the threshold for firing. tRNS might only amplify neural activity that was already present before stimulation, while tACS can interfere with ongoing neural oscillations and change their frequency. However, it is a simplified picture, and recent experiments have revealed that effects of tES are nonlinear and cross-frequency interactions occur. Furthermore, please note that oscillations in the local field potential at the network level can be visible as rhythmic postsynaptic potentials in single neurons. These postsynaptic potentials can already drive neuronal firing and therefore keep the neuron already in a suprathreshold state that might not be affected by external stimulation. In the future, intracranial recordings of neural activity in patients during tES could shed light on many open questions. In addition, a direct comparison of transcranial stimulation methods is desirable, for example, comparing rTMS to tES but also comparing different tES methods with each other (e.g., [62]).

## Figures and Tables

**Table 1:** Translating a current strength of tDCS/tACS (left column) into a voltage gradient (right column) that is used in animal studies. At first, an intracranial current density must be computed in a simulation. If this is multiplied by the gray matter resistivity it yields the voltage gradient.

	tDCS/tACS intensity	Intracranial current density	Gray matter resistivity	Intracranial voltage gradient
Unit	mA	A/m^2^	Ωm	V/m
Value	1	0.1	3.03	0.303

## Additional Points

*Coherence*. Generally, coherence is a measure of the variability of time differences between two time series. “EEG coherence” is often interpreted as a measure of the functional interplay between two brain regions. This can be defined by the direct relationship between the time and frequency domains of the brain oscillations. If the time difference as a phase difference (or angle) is constant over time, the coherence is close to 1.0, and if time difference varies in time from moment to moment then the coherence is closer to 0.* Phase-Locked Activity*. Phase-locked activities contain evoked oscillations that are rigidly time-locked to the moment of stimulus delivery.* Neuronal Entrainment*. It is a term used to describe the property of brain oscillations, how they synchronize their periodicity and rhythm through interaction(s). During this process the given frequency of oscillations resulting from the synchronous electrical activity of neuronal ensembles could be synchronized to the periodic activity of, for example, an internal or external stimulus or event.* Stochastic Resonance*. It is a process that results in an improvement of detection for subthreshold signals by application of noise. It plays a very important role in nonlinear systems, especially in the information processing in the brain.
